# Identification of Six microRNAs as Potential Biomarkers for Pemphigus Vulgaris: From Diagnosis to Pathogenesis

**DOI:** 10.3390/diagnostics12123058

**Published:** 2022-12-06

**Authors:** Wenxiu He, Yixiao Xing, Chunlei Li, Peiru Zhou, Xiaosheng Hu, Hong Hua, Pan Wei

**Affiliations:** 1 Department of Oral Medicine, Peking University School and Hospital of Stomatology & National Center of Stomatology & National Clinical Research Center for Oral Diseases & National Engineering Laboratory for Digital and Material Technology of Stomatology & Beijing Key Laboratory of Digital Stomatology & Research Center of Engineering and Technology for Computerized Dentistry Ministry of Health & NMPA Key Laboratory for Dental Materials, Beijing 100081, China; 2 Department of Stomatology, Zhongshan Hospital, Fudan University, Shanghai 200032, China

**Keywords:** biomarker, diagnosis, microRNA, pathogenesis, pemphigus vulgaris

## Abstract

Background: Pemphigus vulgaris (PV) is a potentially fatal autoimmune bullous disease. The role of microRNA (miRNA, miR) in the diagnosis and pathogenesis of PV remains unknown. This study aims to provide potential miRNA biomarkers for PV diagnosis and therapy options. Methods: Serum samples were obtained from 22 PV patients, 15 mucous membrane pemphigoid (MMP) patients, and 10 normal controls (NC). Total RNA was extracted from the serum samples, and 12 selected miRNAs were detected by quantitative real-time polymerase chain reaction (qRT-PCR). Bioinformatic analyses including target gene prediction and enrichment analysis were performed. Results: Twelve miRNAs were increased in the serum of the PV group compared with the NC group, in which six miRNAs had good efficacy to diagnose PV from MMP with the area under the receiver operator characteristic curves of 0.970 to 0.988. A series test for the combination of miR-584-5p and miR-155-5p reached the sensitivity and specificity of 95.5% and 100%. Bioinformatic analysis revealed target gene enrichment in the cell adhesion pathways, immune-relating pathways, and P38 mitogen-activated protein kinases signaling pathway. Conclusion: The study provides new insights and targets of miRNAs for the precise diagnosis and the exploration of pathogenesis for PV, which may serve as a reference for further research into autoimmune bullous diseases.

## 1. Introduction

Pemphigus are potentially life-threatening autoimmune bullous disorders, in which pemphigus vulgaris (PV) accounts for up to 65% [1]. The prevalence for PV varies from 52 to 95 per million individuals according to ethnicity and regions, and there is a female predominance, with a male-to-female ratio of 1:1.7 to 1:1.1 [2,3]. The pathogenesis of PV is that autoantibodies mainly disrupt desmoglein (Dsg) 3 and Dsg1, leading to the loss of cell adhesion and inducing acantholysis [4]. First-line treatment including corticosteroids and anti-CD20 antibody rituximab have achieved good therapeutic effects for PV [5,6]. However, some adverse effects are associated with the process of long-term therapy, such as Cushing syndrome, infections, and acute infusion reactions, which may even lead to death in severe cases [7,8]. Therefore, it is essential to investigate the biomarkers for concise diagnosis and potential underlying therapeutic targets for a more precise treatment strategy for PV.

MicroRNAs (miRNAs) are single stranded endogenous noncoding RNAs with the length of about 22 nucleotides and serve as the potential regulator of more than 60% coding genes [9]. In the human genome, there are 2654 mature miRNAs according to the miRNA databases miRBase 22.1. MiRNAs bind to the target messenger RNAs (mRNAs) to induce mRNAs’ degradation or translational suppression and subsequently regulate various biological processes and pathways including apoptosis, cell differentiation, intercellular communication, and immune homeostasis [10]. In autoimmune diseases, miRNA dysregulation plays a significant role in disease development. MiRNAs participate in the autoimmune pathogenesis through promoting T cell autoreactivity, activating inflammatory pathways and cytokine production, regulating the activation of autoreactive B cells, and inducing cell apoptosis [11,12]. For rheumatoid arthritis (RA), serum expression levels of miR-194, miR-210, and miR-432-5p have significant differences between the active patients and those in remission. In addition, serum miR-432-5p level is much higher in the RA patients relapsed and the serum level of miR-155 expression is associated with the disease activity of RA. The miRNAs in RA have provided some new biomarkers for the diagnosis and monitoring options of autoimmune diseases [13]. Some studies have revealed miRNAs’ expression profiles in autoimmune blistering diseases. For example, miR-424-5p is overexpressed in the peripheral blood mononuclear cells of pemphigus patients; miR-99, miR-125b, and miR-181 are increased in the keratinocytes of Hailey–Hailey disease (HHD) lesions; some miRNAs, including miR-27a-5p, miR-223, miR-379, miR-423-5p, and miR-1291, are significantly altered in pemphigoid diseased [14]. However, the studies have mainly focused on pemphigus foliaceus, paraneoplastic pemphigus, HHD or pemphigoid diseases, and there are some knowledge gaps regarding miRNAs in PV, especially in the mucosal-dominant variant.

Therefore, this study was designed to provide potential biomarkers to assist PV diagnosis, to investigate the biological significance of miRNAs, and to provide pathogenesis insights for PV.

## 2. Materials and Methods

### 2.1. Study Design and Ethics

This study was designed based on the Standards for Reporting Diagnostic Accuracy (STARD) statement [15]. Ethical approval for this study was obtained from the Ethics Committee of the Peking University School of Stomatology (PKUSSIRB-202162030). All participants provided informed consent before they participated in this study.

### 2.2. Diagnostic Criteria

PV was diagnosed by the Japanese guidelines (2014) based on the clinical features, histopathological presentations, and serologic tests of an enzyme-linked immunosorbent assay (ELISA) or indirect immunofluorescence [1]. Mucous membrane pemphigoid (MMP) was diagnosed based on the histopathological features and positive results of BP180/BP230 by the ELISA tests [16].

### 2.3. Inclusion and Exclusion Criteria

Adult patients (aged 18–75 years) who met the above diagnostic criteria were included in the group of PV or MMP, as determined by experts in oral medicine. Normal controls (NC) were adults without systemic diseases or medication history. Participants were excluded if (A) they had history of topical/systemic use of corticosteroids or immunosuppressors; (B) they were pregnant or lactating females; or (C) they could not cooperate for any reason. According to the inclusion and exclusion criteria, serum samples were collected from 10, 15, and 22 normal controls, confirmed MMP patients, and confirmed PV patients, respectively, from April 2018 to January 2022.

### 2.4. Serum Sample Collection and Processing

Peripheral blood samples from PV and MMP patients and the NC group were obtained by venipuncture and were immediately processed by centrifugation at 1000× *g* for 5 min at 4 °C. The collected serum samples were transported to the laboratory and stored at −80 °C until use.

### 2.5. RNA Extraction, Reverse Transcription, and Quantitative Real-Time Polymerase Chain Reaction (qRT-PCR)

We extracted total RNA from serum samples using the miRNeasy Serum/Plasma Kit (Qiagen, Germany) according to the manufacturer′s instructions. RNA yield was determined with a NanoDrop 2000 spectrophotometer (Thermo Scientific, Waltham, MA, USA), and integrity was determined using agarose gel electrophoresis with ethidium bromide staining.

There was a total volume of 10 μL for each reverse transcription (RT) reaction, consisting of 0.5 μg RNA, 5 μL of 2 × TS miRNA Reaction Mix, and 0.5 μL of TransScrip miRNA RT Enzyme Mix. We carried out the reactions in GeneAmp PCR System 9700 (Applied Biosystems, Carlsbad, CA, USA) at 37 °C for 60 min, followed by RT heat inactivation at 85 °C for 5 s. At −20 °C, 10 μL of the RT reaction mix was diluted 10 times in nuclease-free water. LightCycler 480 II (Roche, Swiss) was used for real-time PCR.

The real-time PCR Instrument (Roche, Swiss) consisted of the 10 μL PCR reaction mixture, including 5 μL of 2 × PerfectStartTM Green qPCR SuperMix, 1 μL of cDNA, 0.2 μL of universal primer, 0.2 μL of miRNA-specific primer, and 3.6 μL of nuclease-free water. In a 384-well optical plate (Roche, Swiss), reactions were incubated for 30 s at 94 °C, then by 45 cycles for 5 s at 94 °C and for 30 s at 60 °C. Analyses were performed on each sample in triplicate. For the validation of the specific generation of the expected PCR product, melting curve analysis was performed after each PCR cycle. The miRNA-specific primer sequences were designed and synthesized by TsingKe Biotech (Beijing, China) (Table 1). The expression levels of miRNAs were normalized to 5S rRNA and were calculated using the 2^−ΔΔCt^ method [17].

### 2.6. Target Gene Enrichment Analysis

The target genes of miRNAs were obtained from four databases including miRDB, miRTarBase, miRWalk, and TargetScan. MiRNA–target mRNA networks and miRNA–target mRNA–pathway networks were conducted using Cytoscape 3.7.1 software (Washington, DC, USA). Gene Ontology (GO) and the Kyoto Encyclopedia of Genes and Genomes (KEGG) enrichment analysis were performed using the GO database and KEGG database [18,19]. The target gene network was mapped using Metascape database [20].

### 2.7. Statistical Analysis

Statistics were analyzed by the Wilcoxon test or *t* test for two independent samples and by one-way ANOVA for more than two samples using SPSS 20.0 software (Chicago, IL, USA). Diagnostic parameters, receiver operating characteristic (ROC) curves, and area under the ROC curve (AUC) were performed using SPSS 20.0. *p* < 0.05 was considered statistically significant.

## 3. Results

### 3.1. Demographic and Clinical Characteristics

The demographic and clinical information of the PV group (*n* = 22), MMP group (*n* = 15), and NC group (*n* = 10) were recorded and analyzed, as shown in Table 2. There were no statistically significant differences in sex and age between the three groups (*p* > 0.05). The 22 patients of the PV group were all mucosal-dominant variant; the mean titers of the Dsg3 and Dsg1 autoantibodies were 148.39 U/mL and 40.29 U/mL, respectively. The Pemphigus Disease Area Index (PDAI) score of the 22 PV patients varied from 1 to 21 points, in which 7 patients had the mild type, and 15 patients had the moderate type. The grading criteria of PDAI were: mild (0–8), moderate (9–24), and severe (≥25) [21].

### 3.2. MiRNAs Expression Profiles

**Figure 1:**
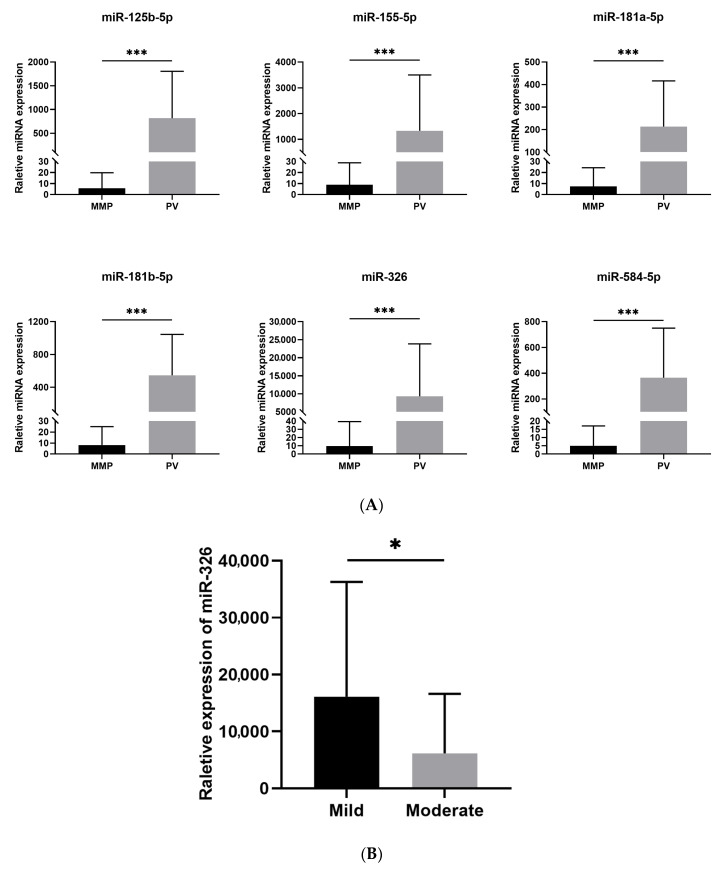
(**A**) Relative expressions of the six selected miRNAs in the PV and MMP groups. (**B**) Relative expression of miR-326 in mild and moderate PV patients. The values represent the mean ± SD. * *p* < 0.05; *** *p* < 0.001.

**Figure A1:**
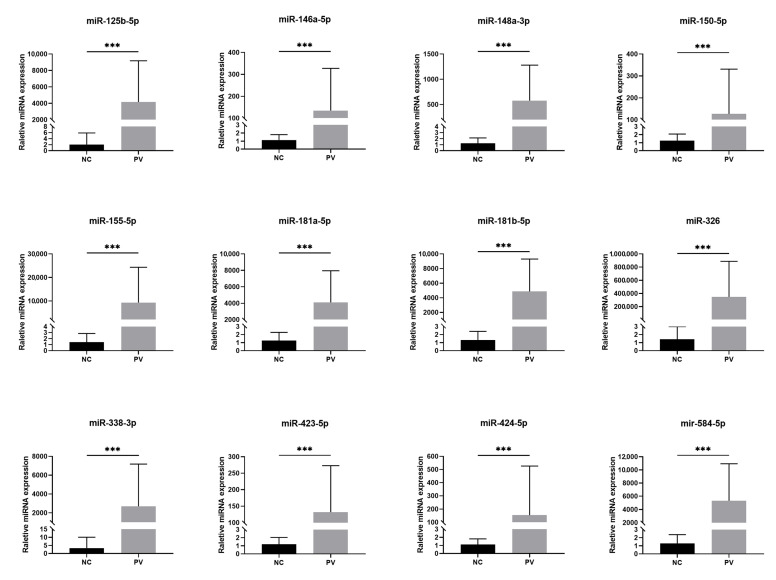
Relative expressions of 12 miRNAs in the PV and NC groups. *** *p* < 0.001.

Twelve miRNAs (miR-125b-5p, miR-146a-5p, miR-148a-3p, miR-150-5p, miR-155-5p, miR-181a-5p, miR-181b-5p, miR-326, miR-338-3p, miR-423-5p, miR-424-5p, and miR-584-5p) were selected based on recent studies about autoimmune diseases and miRNAs [14,22,23]. The 12 miRNAs were detected by qRT-PCR in the PV and NC groups. As illustrated in Figure A1, all 12 miRNAs showed significant upregulation in the PV group (*p* < 0.001), in which the 6 miRNAs with the highest relative expression (miR-125b-5p, miR-155-5p, miR-181a-5p, miR181b-5p, miR-326, and miR-584-5p) were selected for further investigation by qRT-PCR in the MMP group. The expression levels of the six miRNAs were significantly increased in the PV group compared with the MMP group (*p* < 0.001) (Figure 1A). In addition, the difference in miR-326 expression between the mild and moderate PV patients was found to be statistically significant (*p* < 0.05) (Figure 1B).

### 3.3. Diagnostic Efficacy

**Figure 2:**
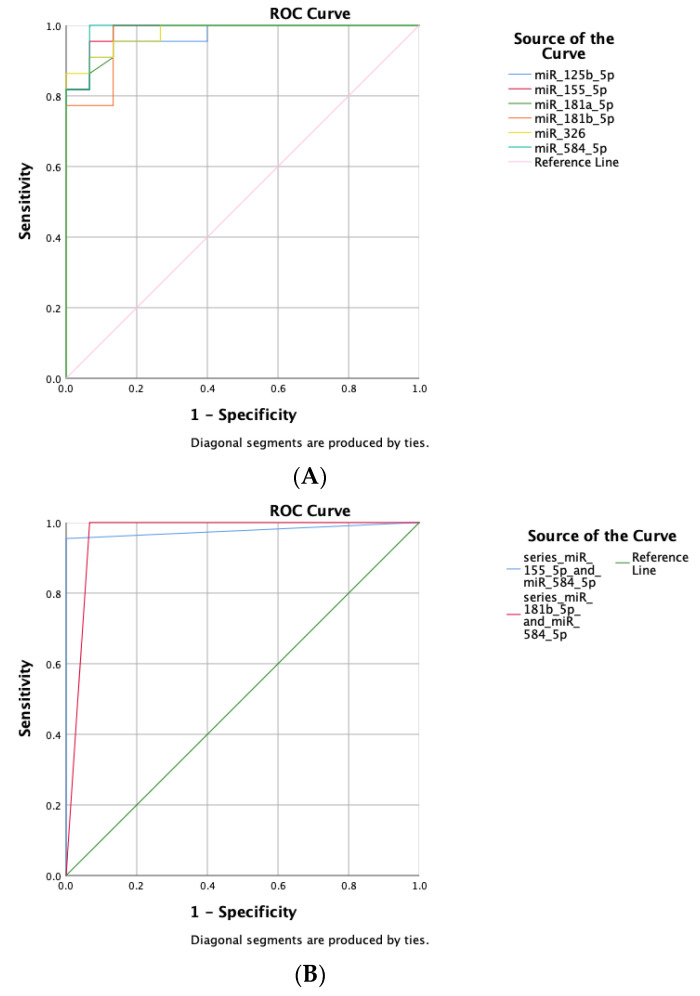
The ROC curves of the miRNAs. (**A**) The ROC curves of the individual miRNAs. (**B**) The ROC curves of the combination of miRNAs.

For the diagnosis of PV, the sensitivity and specificity of miR-125b-5p, miR-155-5p, miR-181a-5p, miR181b-5p, miR-326, and miR-584-5p were 81.8% to 100% and 86.7% to 100%, respectively. The parameters reflecting the diagnostic efficacies are listed in Table 3. The ROC curves of the six miRNAs (Figure 2A) were close to the upper left corner, and the calculated AUC and cutoff values are shown in Table 3, which indicated the good diagnostic efficacy of the miRNAs. To further improve the diagnostic accuracy, the combining of multiple test series was applied. The series test for the combination of miR-584-5p with miR-155-5p reached a sensitivity and specificity of 95.5% and 100%, respectively; the combination of miR-584-5p with miR181b-5p reached a sensitivity and specificity of 100% and 93.3%, respectively. The ROC curves of the series test are shown in Figure 2B.

### 3.4. Targets Prediction for miRNAs

**Figure A2:**
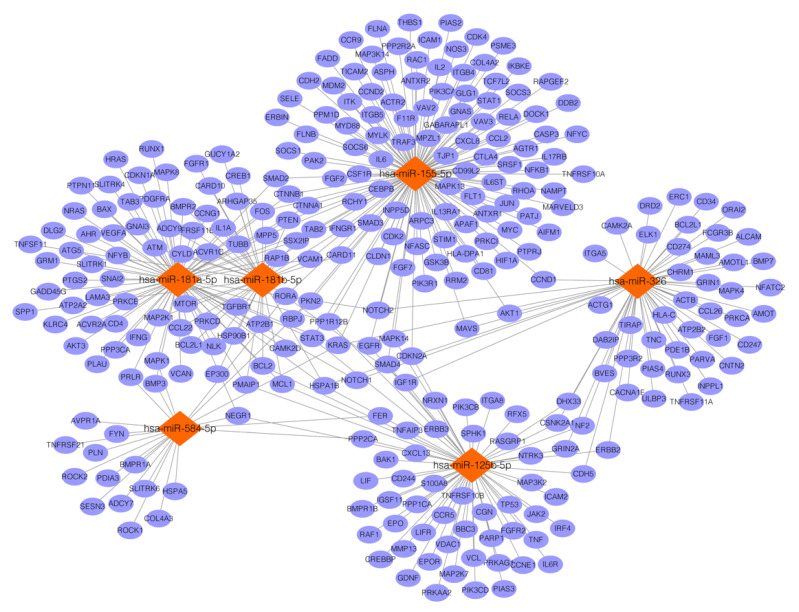
miRNAs–target mRNAs networks of the six upregulated miRNAs in PV.

Four databases, miRDB, miRTarBase, miRWalk, and TargetScan, were used to predict the target genes of miR-125b-5p, miR-155-5p, miR-181a-5p, miR181b-5p, miR-326, and miR-584-5p, and the intersectant results of several databases were used as the candidate target genes of the miRNAs. When the target genes were not detected by both databases, the prediction results from the miRTarBase were used (Table 4). In total, 3411 candidate target genes of the six miRNAs were obtained using the above four databases. The potential PV-relating target mRNAs found by the screening are shown in the network in Figure A2.

### 3.5. GO Analysis

**Figure 3:**
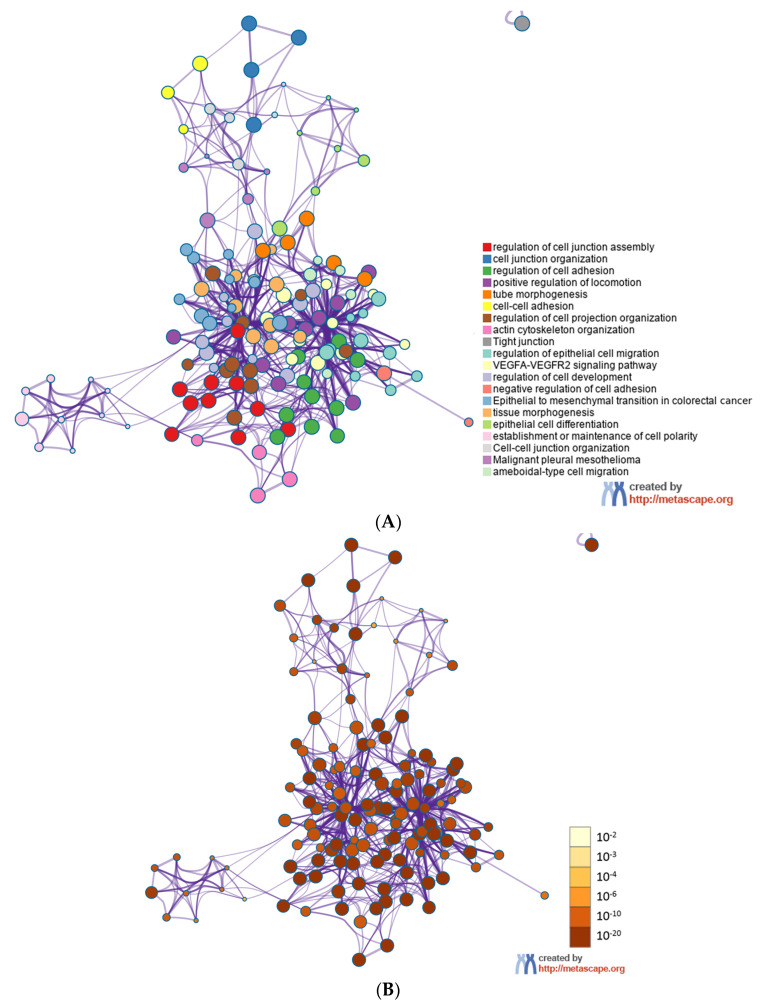
The 133 desmosomes and cell–cell junctions-related target genes were integrated in the network, (**A**) colored by cluster ID, where nodes that share the same cluster ID are typically close to each other, and (**B**) colored by *p* value, where terms containing more genes tend to have a more significant *p* value.

**Figure A3:**
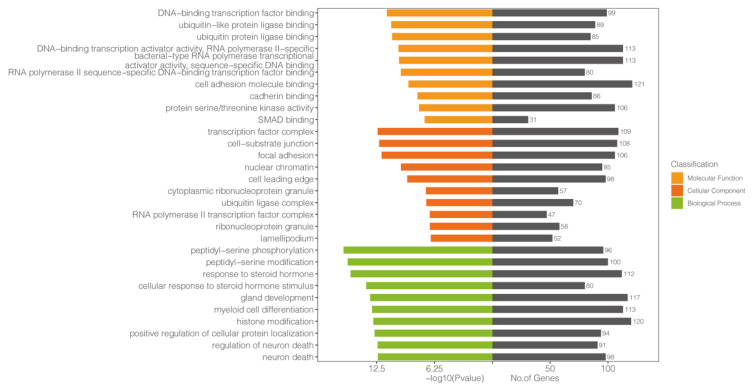
GO enrichment of molecular function, cellular component, and biological process corresponding to the three colors. The vertical axis represents the terms; the horizontal axis represents the −log_10_ (*p* value) and the number of genes.

GO analysis provided significant enrichments of 235 terms in the molecular function category, 195 terms in the cellular component category, and 2267 terms in the biological process category. Figure A3 shows the top ten terms of each category (*p* < 0.05). As for the cellular component, the target genes were markedly enriched in the transcription factor complex, the cell–substrate junction, and focal adhesion. The top two enrichments were DNA-binding transcription factor binding and ubiquitin-like protein ligase binding in the molecular function with peptidyl-serine phosphorylation and peptidyl-serine modification in the biological process. In addition, we integrated 133 desmosomes and cell–cell junctions-related target genes of the miRNAs and mapped the enrichment. The results showed the top three enriched terms were the regulation of the cell junction assembly, the cell junction organization, and the regulation of cell adhesion. Circle nodes with different colors in Figure 3A,B represent the various terms and the *p* values, where size is proportional to the number of input genes that fell under that term. Terms with a similarity score of >0.3 were linked by an edge, and the thickness of the edge represents the similarity scores.

### 3.6. KEGG Analysis

**Figure 4:**
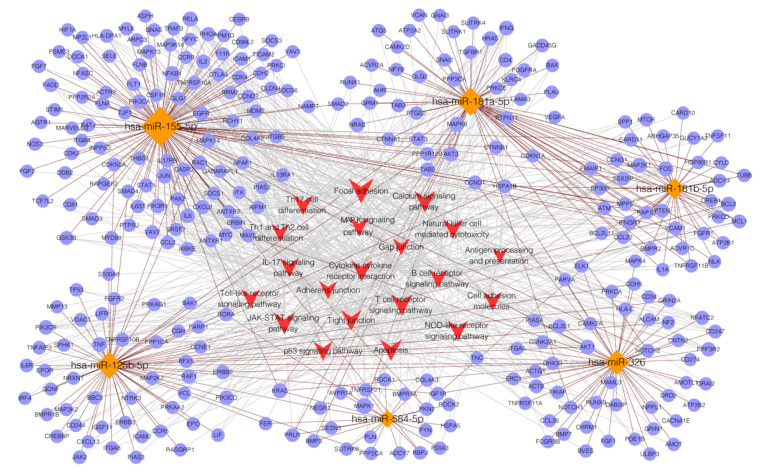
The network of miRNAs–target mRNAs pathways.

**Figure A4:**
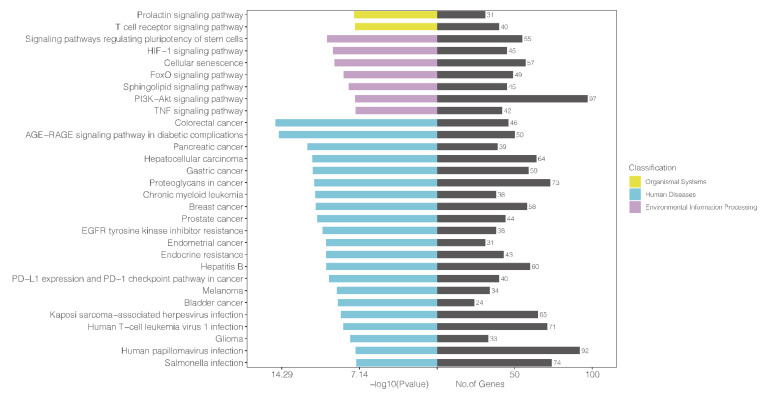
The top 30 terms of the KEGG pathway analysis of the target genes. The vertical axis represents the terms; the horizontal axis represents the −log10 (*p* value) and the number of genes.

Enrichment analysis of the KEGG analysis included 132 KEGG pathways (*p* < 0.05), from which the top 30 terms are shown in Figure A4. In organismal systems, the enriched pathways were the prolactin signaling pathway and the T cell receptor signaling pathway. In the category of environmental information processing, the top three processes of the target genes were the signaling pathways regulating the pluripotency of stem cells, HIF-1 signaling pathways, and cellular senescence. Figure 4 shows the network of the miRNAs–target genes pathways, in which the target mRNAs of the six upregulated miRNAs were remarkably enriched in the pathways relating to the immune system and cell–cell junctions, as well as adhesions, in which the most significant enrichment pathways were the T cell receptor signaling pathway and the p53 signaling pathway.

### 3.7. Adverse Events

There were no severe adverse events in this study.

## 4. Discussion

Pemphigus vulgaris is a potentially fatal autoimmune bullous disease. With the availability and wide use of corticosteroids, the mortality rate of PV has decreased greatly; however, the mortality risk of PV patients is 2.4 to 3.3 times higher compared with that of the general population [24,25,26]. Another first-line treatment of PV is the anti-CD20 monoclonal antibody rituximab, which depletes CD20+ B cells and eliminates the immunoglobulin G (IgG) autoantibodies of PV, reaching disease remission effectively [5,27]. Although the target treatment of rituximab has good efficiency for PV, it brings rituximab-related adverse events including acute infusion reactions (12%), infections (10%), and acute pneumonitis (1%), which may lead to death in severe cases [8]. Thus, the accurate diagnosis, precise therapy, and long-term follow-up are of great significance for PV. This study was designed to evaluate the diagnostic values of miRNAs for PV and explore the bioinformatic predictions of the potential pathways of the differentially expressed miRNAs.

In this study, the sensitivity and specificity of the combination of miR-584-5p and miR-155-5p for the series diagnosis reached 95.5% and 100%, respectively, and the combination of miR-584-5p and miR181b-5p reached 100% and 93.3%, respectively; the AUC of the six miRNAs including miR-125b-5p, miR-155-5p, miR-181a-5p, miR181b-5p, miR-326, and miR-584-5p were 0.970 to 0.988, which indicated the high diagnostic values of miRNAs for PV. As one of the most common techniques in the laboratory, measuring the expression of miRNAs using the qRT-PCR method could be considered to serve as a serologic test for PV diagnosis in clinical practice. In addition, 20–22.7% of the confirmed PV patients were negative for anti-Dsg3 or 1 autoantibodies detected by ELISA [28]. Whether the miRNAs can serve as the complementary approach to ELISA for PV diagnosis and as long-term monitoring biomarkers or not both deserve further research in the future. Currently, there are no well-recognized indicators to monitor the disease activity for PV in long-term follow-up. For miR-326, the expression was much higher in the moderate compared to the mild type for PV, which reflected the disease severity. With the limitation of the small sample size of 7 mild and 15 moderate PV patients, miR-326 could be the potential biomarker for clinical long-term monitoring for PV, while its reliability and stability need further validations with large samples.

To explore the miRNAs’ functions in the pathogenesis of PV, the target mRNAs were predicted using the four databases of TargetScan, miRanda, CLIP-seq, and miRDB. For the hundreds of target genes of the six upregulated miRNAs, NOTCH1, SOCS3, and BCL2 may have significant integration with the pathogenesis of PV. NOTCH1 is the shared target mRNA of miR-181a-5p and miR-326 and is a crucial mediator for the homeostasis of keratinocytes, which was found to be suppressed by miR-125b and decreased in Hailey–Hailey disease [29,30]. The decreased level of NOTCH1 was related to the oxidative stress of keratinocytes, which contributed to the disease activity of pemphigus vulgaris [31]. SOCS3 plays an important role in immune homeostasis. A recent study by Lin et al. showed that the decreased expression of SOCS3 in CD4+ T cells exacerbated the disease severity of PV, and the upregulation of SOCS3 achieved disease remission in turn [32]. BCL2 is the potential target of miR-125b-5p, miR-181a-5p, and miR-181b-5p, is essential for maintaining epithelial integrity, and suppresses Th17 cells [33]. Bcl-2 proteins decreased after PV-IgG treatment in vitro, which contributed to cell death and blister formation [33,34]. Therefore, the six high expressed miRNAs may be involved in the pathogenesis and progression of PV by regulating the target genes’ expression profiles.

The bioinformatic analysis showed the target genes of the miRNAs enriched in the pathways of cell adhesion molecules and some extracellular junctions. In addition to the well-recognized autoantigens of the extracellular domain of Dsg 3 and 1, there are other potential autoantigens belonging to desmosomes including Dsg 2 and 4, desmocollins, plakophilin, E-cadherin, and plakoglobin [35,36]. Some studies have reported antigens of the adherens junction components such as α-catenin, suggesting that the early triggering of acantholysis in PV was mediated by non-Dsg autoantibodies, and the pathogenesis of PV was contributed to by Dsg 1/3 and non-Dsg antibodies together [37]. Bioinformatic analysis also indicated the enrichment of immune-relating pathways such as Th1, Th2, and Th17 cell differentiation. The increased reactivity of Th1 and Th2 cells were both detected in PV pathogenesis [38]. Autoreactive Th2 cells were crucial for the production of anti-Dsg3 antibodies in vivo, which contributed to the pathogenic autoantibody secretion of B cells, and were related to the disease activity of PV [39,40]. The level of Th17 cells were detected as increased in both the circulation and PBMCs, as well as the lesions of PV patients [41,42,43,44]. In addition, the inflammatory cytokines of IL-10 and IL-17 associated with Th2 and Th17 cells showed marked elevation in PV serum, which participate in the pathogenesis and activation of PV [45]. T cell subsets play key roles in immune regulations and may induce some future therapeutic options for PV, which agreed with our bioinformatics analysis. P38 mitogen-activated protein kinases (MAPK) signaling, one of the significant enrichment pathways in our bioinformatic results, has been reported to be activated in PV pathogenesis and plays an important role in intercellular adhesion [46,47]. The p38MAPK activation process induced by PV autoantibodies was regulated by keratin filaments, indicating the complicated cell adhesion networks involved in the pathways and disease pathogenesis [46]. Currently, there is no p38MAPK targeted inhibition therapy available for the management of PV; the therapeutic targets of p38MAPK may deserve further investigation and exploration [48,49].

There were several limitations in the present study. Firstly, as an exploratory study, the sample size was small and without follow-up observation. The current results and the values of follow-up biomarkers for the miRNAs need to be confirmed by further studies with larger groups and long-term monitoring. Secondly, further experiments are necessary to confirm the results predicted by the bioinformatics analysis. In conclusion, this study filled the gap about miRNAs in PV diagnosis and pathogenesis. The results provided new insights for precise diagnosis and new targets for the pathogenesis of PV, which may serve as references for the future research of PV and autoimmune bullous diseases.

## Figures and Tables

**Table 1:** Primer sequences of qRT-PCR.

miRNAs	Forward Primer Sequence (5′-3′)
hsa-miR-125b-5p	CTGAGACCCTAACTTGTGAAA
hsa-miR-146a-5p	AGAACTGAATTCCATGGGTTAA
hsa-miR-148a-3p	AGTGCACTACAGAACTTTGTAA
hsa-miR-150-5p	CCCAACCCTTGTACCAGTG
hsa-miR-155-5p	TTAATGCTAATCGTGATAGGGG
hsa-miR-181a-5p	TTCAACGCTGTCGGTGAGTAA
hsa-miR181b-5p	ATTCATTGCTGTCGGTGGGT
hsa-miR-326	TGGGCCCTTCCTCCAGAAA
hsa-miR-338-3p	CAGCATCAGTGATTTTGTTGAA
hsa-miR-423-5p	GGGCAGAGAGCGAGACTTT
hsa-miR-424-5p	CAGCAGCAATTCATGTTTTGAA
hsa-miR-584-5p	GTTTGCCTGGGACTGAGAAA
5S	GGAGACCGCCTGGGAATA

**Table 2:** Demographic and clinical characteristics.

Group	Sex (Female/Male)	Age (Mean ± SD)	ELISA (U/mL)		PDAI Score (Mean ± SD)	Disease Severity	
			Dsg3 (Mean ± SD)	Dsg1 (Mean ± SD)		Mild (*n*)	Moderate (*n*)
PV group (*n* = 22)	12/10	53.23 ± 8.74	148.39 ± 33.84	40.29 ± 42.71	11.09 ± 6.21	7	15
MMP group (*n* = 15)	10/5	58.80 ± 10.65	-	-	-	-	-
NC group (*n* = 10)	6/4	53.50 ± 9.09	-	-	-	-	-

**Table 3:** Diagnostic efficacy of miRNAs for PV.

miRNAs	AUC			Cutoff	Youden Index	Sensitivity (%)	Specificity (%)	PPV (%)	NPV (%)
	Area	SE	95% CI						
miR-125b-5p	0.970	0.024	0.923–1.000	8.575	0.822	95.5	86.7	91.3	92.9
miR-155-5p	0.985	0.015	0.955–1.000	39.025	0.888	95.5	93.3	95.5	93.3
miR-181a-5p	0.980	0.018	0.946–1.000	67.150	0.818	81.8	100	100	78.9
miR181b-5p	0.970	0.025	0.922–1.000	15.835	0.867	100	86.7	91.7	100
miR-326	0.979	0.018	0.943–1.000	178.440	0.864	86.4	100	100	83.3
miR-584-5p	0.988	0.014	0.960–1.000	10.455	0.933	100	93.3	95.7	100
miR-155-5p + miR-584-5p	0.977	0.026	0.925–1.000	-	0.955	95.5	100	93.8	100
miR-181b-5p + miR-584-5p	0.967	0.038	0.892–1.000	-	0.933	100	93.3	100	95.7

**Table 4:** The numbers of target genes of miR-125b-5p, miR-155-5p, miR-181a-5p, miR181b-5p, miR-326, and miR-584-5p predicted by the four databases (TargetScan, miRanda, CLIP-seq, and miRDB).

miRNAs	miRDB	miRTarBase	miRWalk	TargetScan	Candidate Target Genes
miR-125b-5p	925	432	6148	1736	622
miR-155-5p	701	904	2842	3296	1030
miR-181a-5p	1408	553	3106	3363	727
miR181b-5p	1408	375	5335	290	408
miR-326	759	138	7930	3317	391
miR-584-5p	402	67	7670	2667	233

## Data Availability

The data presented in this study are available on request from the corresponding authors.
